# Geospatial Assessment of Cholera in a Rapidly Urbanizing Environment

**DOI:** 10.1155/2017/6847376

**Published:** 2017-04-11

**Authors:** Olajumoke Esther Olanrewaju, Kayode Adewale Adepoju

**Affiliations:** Institute of Ecology and Environmental Studies, OAU, Ile-Ife, Osun State, Nigeria

## Abstract

This study mapped out and investigated the spatial relationship between cholera incidences and environmental risk factors in the study area. The study area was stratified into eight zones. Water samples from each zone were collected and analyzed to determine the colony forming units. GIS layers including housing density, digitized roads, rivers, buildings, and cholera incidence data from hospital archives were also collected and analyzed using ArcGIS 10.1. It was observed that there was an association between the ERFs (*p* < 0.001). Similarly, 18 out of the 44 waste dump sites, seven out of 18 markets, and two out of 36 abattoirs were found near the historical cholera cases. Similarly, 4 (21.1%) locations were traced to be predominantly close to rivers and waste dump site. All the historical cholera cases were found adjoining to roads and buildings. Highest CFU count was found in the wells and streams of areas with a cluster of all the environmental risk factors and high housing density. This study revealed that waste dump sites and market had the highest predisposing attribute while the least was abattoir. The uniqueness of the study lies in the combination of mapping and microbial analyses to identify and assess the pattern of cholera risk and also to provide clear information for development of strategies for environmental supervision.

## 1. Introduction

Cholera is an acute intestinal infection caused by the ingestion of food or water contaminated with the bacterium* Vibrio cholerae* [1]. Cholera has a short incubation period, from a few hours to 5 days, but commonly 1-2 days [2]. Cholera affects all ages and both genders [2]. The bacterium can live naturally in any environment especially in brackish rivers and coastal waters [3]. Human behaviours related to environmental hygiene, personal hygiene, and food preparation contribute greatly to the occurrence and severity of cholera [4]. Transmission is usually due to fecal contamination of food and water as a result of poor hygiene. Inappropriately managed wastes can attract rodents and insects, which can harbor gastrointestinal parasites, yellow fever virus, worms, and the plague pathogen. Wastes dump sites, markets, abattoirs, and vulnerable elements such as rivers, roads, and buildings have been found to be associated with the occurrence of diseases [1].

Cholera as a disease is endemic in Africa, parts of Asia, the Middle East, and South and Central America [5]. In endemic areas, outbreaks usually occur when war or civil conflict disturbs public sanitation services. Also, the normal balance of nature is being interrupted by natural disasters like earthquake, tsunami, volcanic eruptions, landslides, and flood [6]. Therefore many health problems are created; food and water supplies can become contaminated by parasites and bacteria when essential schemes like those for water and sewage get compromised. Due to the increasing size of vulnerable populations living under unhygienic and deprived environmental conditions, the numbers of cholera cases reported in developing countries continue to rise [7]. Most of the vulnerable populations remain in locations where access to safe drinking water and good hygiene practices are not in place [5]. Markets, waste dump sites, and abattoirs are major socioeconomic factors that often expose water sources, roads, and residents in their neighborhoods to danger of contamination and disease. In Nigeria, the infection is endemic and outbreaks are not rare. The first series of cholera outbreak was reported between 1970 and 1990 [8]. Additionally, more than 260 individuals were speculated to have died of cholera in four Northern states of Nigeria with over 96 individuals in Maiduguri, Biu, Gwoza, Dikwa, and Jere council areas of Bauchi state [9]. The last outbreak of cholera in Ile-Ife, Osun State, in Nigeria resulted in ill-health of many residents and loss of lives. Despite this long experience with cholera, an understanding of the epidemiology of the disease aiding its persistence in outbreak situations is still lacking. This study was therefore borne out of a need to understand the relationship between the spatial pattern of cholera risk and the associated socioeconomic and environmental factors in affected communities in Ile-Ife.

## 2. GIS and Cholera Mapping

New approaches in geography and related fields, capitalizing on advances in technologies such as geographic information systems (GIS), the global positioning system (GPS), satellite remote sensing, and computer cartography, make it possible to integrate highly accurate geographic locations and time with virtually any observation [10, 11]. GIS provides the means to store, analyze, share, and visualize real time and archived spatial data. It also permits the integration of multiple layers of interdisciplinary spatial data, such as health, environmental, genomic, social, or demographic data for interactive spatial analyses and modeling. Satellite images can greatly enhance mapping of the environmental factors associated with cholera risk just like some other infectious diseases. Integrating satellite images, spatial statistics, and GIS can provide ways of relating health and diseases to specific genetic, epigenetic, and environmental factors [12]. GIS has been used extensively in epidemiology for disease surveillance and intervention monitoring [13–15]. By mapping disease cases in geographic space, local and national governments can easily identify the triggers, distribution, and spread of disease across geographic regions, optimize planning of intervention locations, and monitor their effectiveness.

## 3. Methodology

### 3.1. Study Area

**Figure 1:**
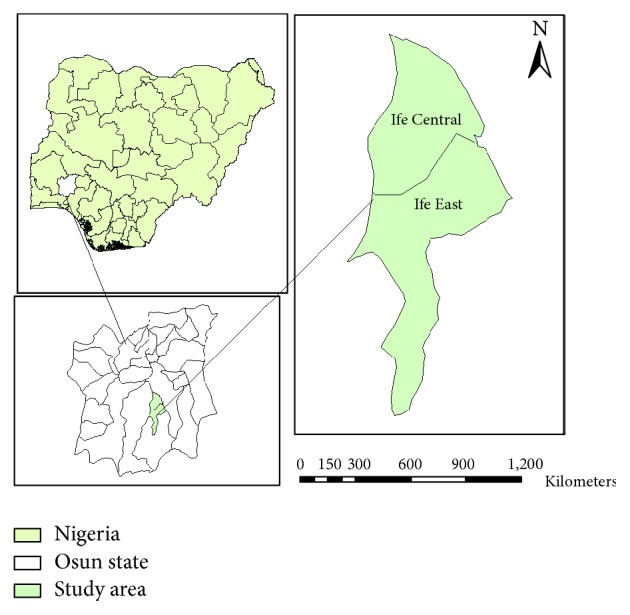
Map of the study area.

Geographically, Ile-Ife is located between Latitude 07°28′N and 07°45′N and Longitude 04°30′E and 04°34′E [16]. The climate is tropical. Like every other Southwest area, the raining season extends from April to October while the dry season lasts from October to March. Ile-Ife has two major local governments, namely, Ife Central and Ife East. The town has been witnessing population and physical growth [17]. As a result of such growth, many abattoirs were created because the meat needs of people are on the increase and more waste dump sites were generated. The study was carried out in Ife East and Ife Central areas of Ile-Ife (Figure 1). Ife East LGA has an area of 172 km^2^ while Ife Central LGA has an area of 111 km^2^. The LGAs populations have tremendously increased over time. Ife East population was 188, 087 as at the 2006 census while that of Ife central population was 167,254 as at 2006.

## 4. Data Acquisition, Sampling Design, and Processing

Primary data utilized for this study was obtained with the use of a global positioning system (GPS) between May and June, 2015, and the environmental risk factors were selected based on WHO predisposing factors to cholera [18]. These include coordinates of abattoirs, waste dump sites, and markets. Altogether 34 abattoirs (A), 42 waste dump sites (WDS), and 18 markets (M) were mapped during this study. The secondary data utilized for this study include recent high resolution image of the study area (Google earth), a land-use/land-cover map, digitized roads, rivers, and buildings. Spatial analysis was carried out to stratify the areas into eight (8) different environmental risk zones based on density analysis using ArcGIS software version 10.1. Cholera incidence data were obtained from Ife East and Ife Central local government areas at the Monitoring and Evaluation Department for the years 2010 and 2011 when the prevalence was very high (Table 1). Spatial analysis tool of proximity function in ArcGIS software 10.1 was used to determine the relationship between cholera incidences and the associated socioeconomic and environmental risk factors. Also, inferential statistics (Chi-square) was used to determine association between the ERFs. A purposive sampling technique was used to determine where water samples were gotten, though the sampling was based on the proximity to the ERFs identified for this study. Therefore, a total number of 59 water samples were collected for this study. Thirty-six water samples were collected from wells while twenty-three water samples were collected from streams. Water samples for microbial analysis were collected in sterile universal bottles. The samples were kept in an ice pack, transported to the laboratory immediately, and some that were not used immediately were kept in the refrigerator to prevent contamination, and this was done according to Golterman et al. [19] and WHO [20] methods for water analysis. Water samples were collected in the morning within the period of 06:30 to 08:30 Nigerian local standard time. Sterile distilled water was placed in test tubes representing the dilution from 10^−1^ to 10^−10^, and serial dilution was done by introducing the samples in the correct proportion to the sterile distilled water. Serial dilutions of 10^−4^, 10^−5^, and 10^−6^ were chosen. Furthermore, 1 mL each of the dilution was poured in the plates and prepared blood agar medium was poured into it. It was thereafter evenly spread throughout the plates according to Chessbrough directive [21]. The plates were thereafter incubated at 37°C for 24 hours after which the colonies were counted and recorded. The degree of contamination of water samples was ranked according to Monica specification [21]. The period for the collection and the laboratory analysis lasted from July to August, 2015. The laboratory analysis of the water samples for* Vibrio cholerae* was done thrice for quality assurance sake. Some tests that were carried out on each isolate to determine the presence of* Vibrio cholerae* after culturing in alkaline peptone water and thereafter subculturing in Thiosulphate Bile Salt Sucrose (TCBS) and a nutrient agar included Sulfide Indole Motility (SIM), Triple Sugar Iron (TSI), citrate utilization test, urease test, and oxidase tests. Results were interpreted using Bergrey's manual of determinative bacteriology [22] to determine the presence of* Vibrio cholerae*.

## 5. Conceptual and Statistical Analyses

The georeferenced cholera cases were plotted on the database map. The distance of each environmental risk zone to the cholera incidences was geovisualized to check the proximity (relationship) between them. The result was presented in the form of a map. Also, proximity analysis which required a buffering of 300 meters from the environmental risk factors to the historical cholera incidences was done with ArcGIS software 10.1. The result of the* Vibrio cholerae* and the CFU count per sample were used to build up the attribute for each of the water sample points on GIS map. The level of contamination was displayed on the map and integrated with the cholera database to produce a composite map of cholera cases housing density and predisposing factors in the study area. The highest CFU count was given >100, followed by 10.1–100, while the lowest count was given the range of 1 to 10 [20, 21]. Spatial analysis was performed to stratify the areas into different disease hot spot density from very high to very low. Descriptive and inferential statistics (Chi-square) was used to interpret the questionnaire. Inferential statistics (Chi-square) was used to determine the association between different variables in the study. All statistics were discussed at 95% confidence level (*p* < 0.05).

## 6. Result

The different rings with intersections depict areas of highest densities of each of the risk factors. The red ring depicts a zone of high concentration of abattoirs, while the yellow ring depicts zones on highest concentrations of markets and the green zone represents areas with highest concentrations of waste dumps. The various intersections between abattoir and market, abattoirs and waste dump, and waste dump and markets are zones of interactions among the factors. Another zone, which is central to all the rings, depicts areas of intersection among abattoir, market, and waste dumps. The other zones that fall outside the rings are areas of low density of environmental risk factors. The rivers and streams flowed through all the designated areas of the environmental risk factors (ERFs). Most of the abattoirs were located around the streams and rivers. Abattoirs were generally found to be located along the river bodies and towards the northwestern part of the study area such as Old-Nepa and Ope-Oluwa. Wastes were generally found to be distributed mainly along the road sides and rivers which were towards the northeastern part of the study area (Ojoyin, Lafogido, and Iyana-Oja). The waste dump sites observed in the study were found to be clustered around the roadside and mainly in the northeastern part of the study area especially around Ojoyin Street, Lafogido, Igbo-Itapa, Ajamopo, and Ile-Lami compounds. These areas are mostly characterized by housing density which is typical of inner city core found in many developing countries. This development can contribute to overcrowding causing the easy spread of diseases.

**Figure 2:**
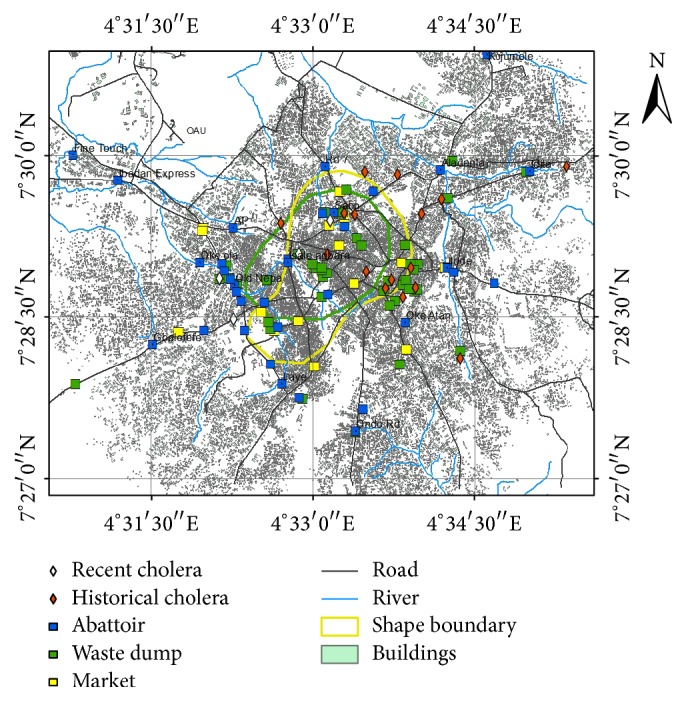
Spatial relationship of cholera incidences and the environmental risk factors.

The 19 historical occurrences of cholera in Ife East and Ife Central LGA in the years 2010 and 2011 can be traced to the presence of markets which contained bulk of waste dump sites proximate to the residential areas (Figure 2). Spatial analysis of 300-meter buffer showed the different hot spots for cholera incidences. It was observed that 18 out of the 44 waste dump sites located in this study were near the historical cholera cases. These areas are Sabo 1, Sabo 2, Sabo 3, Ojoyin 3, Ojoyin 4, Ojoyin, Ayegbaju, Ayegbaju 2, Ilode, Ajamopo, Ile-Lami compound, Ile-Lami, Oke-Atan, Ayetoro/Iloro, Ajamopo 2, Esinmirin, Otutu, and Odi-Olowo. Similarly using the same approach, 7 out of the 18 markets in the study were observed to be near the historical cholera incidences and these areas are Oja Ife, Ojatutun market, Itakogun market, Iso obi market, and Oduduwa street market. Finally, Sabo abattoir and Oduduwa street abattoir were the two (2) abattoirs selected out of 36 abattoirs that were found to be proximal to the historical cholera cases (Figure 2 and Tables 2 and 3). Summarily from this study, waste dump sites and market were identified as the major factors associated with the historical cholera cases. This is because majority of the cholera cases were found proximate to waste dump sites in the zone of market. It was also deduced from this study that there is an interlink between cholera prevalence and proximity to water bodies. The least was the proximity to abattoir. Also,* Vibrio cholerae* were isolated in the stream at Iyana-Oja, Famia road, Ojoyin, and Olanrewaju street.

## 7. Environmental Risk Map for the Study Area

**Figure 3:**
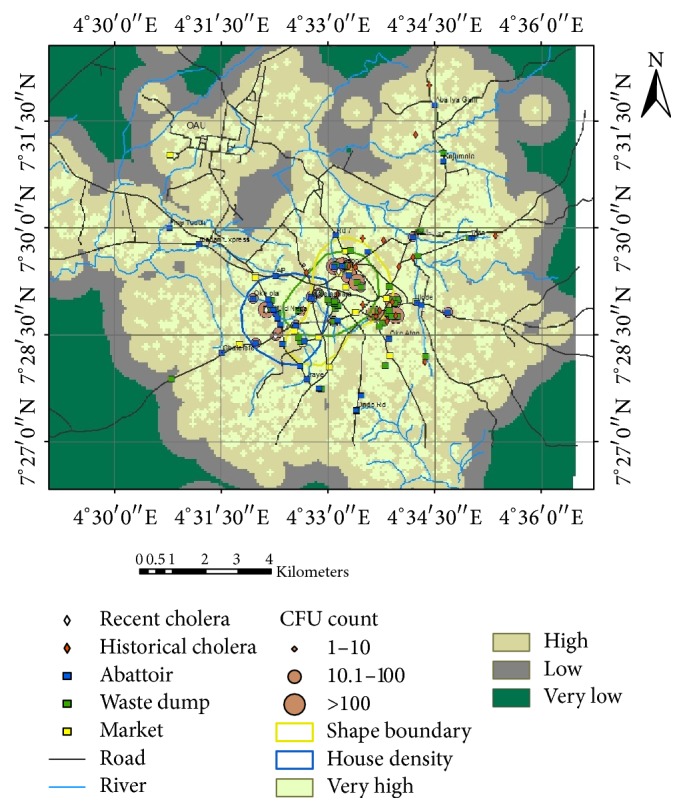
Composite map of cholera cases housing density and predisposing factors in the study area.

When the CFU count was overlaid on housing density layer in ArcGIS, the CFU count was found to decrease in order of reducing magnitude of housing density from high to low. The highest CFU count was found in the wells and streams at Sabo community, Olanrewaju dump site, and Iyana-Oja dump site. High CFU counts were found in Oduduwa street, Ojoyin Street, and Famia Street. This is followed by Better-Life, Ola-Olu, Oduduwa Slum, Olonode, Akaui Street, Lafogido, Igbo-Itapa, and Ile-Lami (Figure 3).

## 8. Discussion

The main socioeconomic factors (markets, waste dumps, and abattoirs) were generally found to be located along water bodies, roads, open lands, and high density neighborhoods. The nature of their proximity could cause contamination along water courses and major roads and also constitute major health challenge to the residents in their neighborhoods especially during flood and other forms of emergencies. Wastes also constitute nuisance to the environment and form the major breeding site for rodents and other diseases causing organisms. These nuisances could also constitute a major health challenge to the residents within the locations because it is likely for the disease to propagate from its origin to proximal communities earlier than communities which are farther away. References [23–25] corroborate these verdicts. Poverty and ineffective provision of adequate waste disposal methods can contribute greatly to the indiscriminate disposal practices in which the indigenous people engaged. Meanwhile, government policies or institutions responsible for community health are usually underfunded or not motivated to control the situation. References [26–28] identified that the occurrence is rampant in many developing countries. A study carried out in the eastern area of Nigeria on proximity of municipal waste and rate of hospitalization for malaria indicated that proximity of waste dump to roads and residential areas has imparted negativity on the health of the public [29].

From this study, waste dump sites were identified as the major factor associated with the historical cholera cases. This is because majority of the cholera cases were found proximate to waste dump sites. Therefore, the cholera outbreak at these places might be due to the high rate of breeding of flies which could serve as a carrier of* V. cholerae* from the waste dump sites polluted by excreta and human garbage to man's food and water. Reference [30] also identified the occurrence of these flies in waste dump sites. Research has also proven that the common housefly (*Musca domestica*) and flies generally are mechanical vectors of many kinds of pathogens such as bacteria, protozoa, viruses, and helminth eggs [31]. Reference [32] undertook a study on vector potential houseflies* (Musca domestica)* in a transmission of* V. cholerae* in India and this study ascertained the vector potential of the domestic housefly as a carrier of* V. cholerae* in Delhi, India, where an outbreak of cholera was encountered. Flooding could occur during the heavy outpour of rain, thereby causing contaminated water to flow into the water sources used for human daily activities. Also, surface run-offs from these dumps sites serve as a major pathway for fecal and bacterial contamination of rivers and streams. The run-offs also carry high organic loads, leading to stagnation and increased salinity of rivers and streams, thus producing appropriate environmentally friendly sites for the cholera* Vibrio*. This was supported in the study conducted on the influence of environmental factors on the presence of* V. cholerae* in the marine environment: a climate link [33]. A research undertaken in Kumasi, Ghana, on the spatial dependency of* V. cholerae* on open space refuse dumps with a spatial statistical modeling suggests that cholera risk is relatively high when inhabitants live in close proximity to waste dumps and where there are numerous refuse dumps [34].

Specifically, the major outbreak of cholera in Ile-Ife which was found to occur at Sabo could be further associated with the presence of abattoir in this community. Ideally, abattoirs should be located few meters away from residential areas and where there would be ample supply of water for cleansing and a means of transporting the treated remnants. However, for Sabo community, the abattoir is located inside the community which made the wastes from this activity to be poorly handled, hence serving as a breeding place for flies which could predispose them to various diseases especially cholera. Also, other environmental factors like markets and waste dump sites were found near this community. This cluster of factors could further enhance the spread once there is an outbreak.

It was also deduced from this study that there is an interlink between cholera prevalence and proximity to water bodies. This is because water bodies are generally being polluted by refuses and human excreta due to the indiscriminate defecation practices of inhabitants inside water bodies. The risk of cholera incidence is assumed to be greater for people who live closer to them and thus will possibly tend to have higher cholera prevalence than those who live farther. This finding was corroborated by the finding of Ali et al., who reported that cholera infection is enhanced by proximity to potentially polluted surface water bodies [35]. Thus, this is also an indication that the effects of dump sites on cholera infection require surface water as an intermediate pathway. Therefore, any attempt to prevent defecation at dump sites will reduce fecal contamination of rivers and streams. This will in turn reduce cholera infection during any outbreak. Also, the epidemiological work of John Snow in London revealed the association between cholera and contaminated water even before any bacteria were known to exist [36]. Odi-Olowo, Mokuro, Seminary Opa, Mount Zion, and Olubuse from the southwestern part of the study area are areas which are positioned near water bodies, and thus the cholera cases in these communities may be attributed to proximity to water bodies.

In a similar manner, proximity to market was identified to be associated with incidences of cholera in the study area. This is because market is associated with bulk of wastes and also some of these markets were found to be proximal to water bodies. These made dumping of wastes inside the nearby streams very easy. The markets which were found to be close to waste dump sites were mostly located in the northeastern part of the study area which has a very high housing density.

More so, market sites were found running parallel to major transportation routes as the sites were characterized with a lot of road networks which make them generally a center of commerce. Also, markets are known to be close to residential areas for easy accessibility. Oja-Tutun market in the study area is the largest market in Ile-Ife and a major center of commerce in the town which lacked adequate sanitation measures especially in the deposition of waste. Due to lack of waste collection systems in this place, people found it convenient to deposit their waste inside the stream close to the market. Cholera outbreaks could have occurred due to the fact that microorganisms found their way to the various waste dump sites around the market. During wet seasons, water could percolate through the soil, carrying these organisms to local surface water, ground water, or sea [37]. Therefore, proximity to the market also plays a significant role in cholera transmission because of the large amount of wastes generated.

The composite cholera risk map produced from this study serves as a prediction to the future outbreak of pathogenic diseases. The high level of contamination of the water sources is due to the presence of pathogenic bacteria and higher colony forming unit (CFU) counts present in them. The reason for the high CFU concentration in Sabo community is because there is a cluster of abattoirs, waste dump sites, and markets around the location. The combination of the three environmental risk factors in the same region further aggravates the situation and makes the areas to be more vulnerable to cholera infection and other bacteria diseases caused by poor sanitation. Other locations with higher CFU counts were Ola-Oluwa, Iyana-Oduduwa, Ojoyin, Olanrewaju, Isale-Agbara, Famia, Better-Life, Akaui, Oja-Titun, Iyana-Oja, Olorunsogo, Olonode, Lafogido, Igbo-Itapa, and Ile-Lami compounds with or without history of cholera incidences. These areas could be predisposed to outbreak of diseases because their CFU count exceeded the WHO permissible limits for drinking water [38, 39]. The work of Obiri-Danso et al. [40] on the spatial dependency of cholera prevalence on potential cholera reservoirs in an urban area in Kumasi, Ghana, deduced that the spatial distribution of cholera prevalence is dependent on higher bacterial counts found in water. This signifies that areas with the highest CFU counts are at higher risk of outbreak of diseases. It was also observed from the study that the new detected cases of* V. cholerae* are spreading towards the northwestern part of the study area. These areas are Famia road, Olanrewaju street, and Ojoyin community and the last one is at the northeastern part of the study area (Iyana-Oja market), in proximity to where cholera outbreak had occurred before. This poses a serious risk to the inhabitants of these communities because any contact with the water source could lead to an outbreak of cholera and other pathogenic diseases. Similarly, the hot spots clusters of cholera cases were seen mainly in areas of very high to high population densities. These areas are Iyana-Oja, Ajamopo, Ile-Lami, Oke-Atan, Igbo-Itapa, Sabo, Iyana-Oduduwa, Oja-Titun, Old-Nepa, Oke-Ola, Ilode, Ojoyin, Aba-Iya Gani, Opa, Kojumole, A.P, and Eleyele. If proper interventions are not taken, the proximity of residential buildings to disease hot spots could play an important role in transmission of the disease to human.

## 9. Conclusion

The spatial relationship between cholera incidences and ERFs generated in this study showed that areas with historical cholera cases were close to waste dump sites near market, rivers/streams, and abattoirs with waste dump sites and markets having the highest predisposing attributes while the least was abattoir. The composite cholera risk map indicated the relationship between historical cholera cases and CFU count (Sabo, Ola-Oluwa, Iyana-Oduduwa, Ojoyin, Olanrewaju, Isale-Agbara, Famia, Better-Life, Akaui, Oja-Titun, Iyana-Oja, Olorunsogo, Olonode, Lafogido, Igbo-Itapa, and Ile-Lami compounds) and medium CFU count and low CFU count. Also, behaviour and socioeconomic conditions may promote the contamination of water with cholera. Sabo is usually associated with cholera contamination for other reasons apart from waste dump site. Based on the findings from this study, the environmental factors identified in this study had a significant pollution effect which could pose a severe risk to the health and wellbeing of users and this calls for urgent intervention.

## 10. Recommendations

Communities such as Ajamopo, Igbo-Itapa, Ile-Lami, Ayegbaju, Ojoyin, Sabo, Ola-Olu, and Iyana-Oduduwa which have waste dump sites proximate to them should be given quick intervention by removing the wastes and providing refuse vans to eliminate such nuisances. Also, communities that are proximal to markets should be properly observed because of the huge amount of wastes markets generate. Also, indiscriminate dumping of wastes should be highly prohibited and anybody that dumps wastes indiscriminately should be sanctioned by health authorities in the region. Also, government should make sure that markets and abattoirs are sited at least 300 meters away from residential areas.

Furthermore, communities such as Seminary Opa, Mokuro, Odi-Olowo, Sabo, and Mount Zion that have rivers and streams close to them should be properly monitored more importantly by creating good drainages for the free-flow of water in order to control them from overflowing to residential buildings which could cause flooding and leads to outbreak of diseases. Also, Sabo community that has an abattoir in her community should be banned from operating by appropriate health authorities in the region.

It is also recommended that water sources in communities such as Sabo, Ola-Oluwa, Iyana-Oduduwa, Ojoyin, Olanrewaju, Isale-Agbara, Famia, Better-Life, Akaui, Oja-Titun, Iyana-Oja, Olorunsogo, Olonode, Lafogido, Igbo-Itapa, and Ile-Lami compounds with higher CFU counts should be treated. Communities such as Ojoyin, Iyana-Oduduwa, Famia, and Olanrewaju with their water sources being contaminated with the presence of* V. cholerae* should be properly monitored to prevent outbreak of diseases. Also, periodic water testing should be undertaken by health authorities in the regions every three months to detect any microorganisms that may be present in order to apply treatment accordingly.

## Figures and Tables

**Table 1:** Locations of reported cholera cases at Ife Central LGA (August, 2010) and Ife East (October, 2011) local government areas.

Ife Central LGA	Long (0E)	Lat (0N)	Ife East LGA	Long (0E)	Lat (0N)
Sabo	004.554862	07.491038	14, Igbo Itapa	004.562142	07.480715
13, Ogbingbin	004.552293	07.484630	29, Lafogido junction	004.561255	07.479310
Seminary opa	004.570465	07.521842	Bk 17, Ajamopo	004.563903	07.477990
Ilare	004.556458	07.490795	26, Ayegbaju street	004.565123	07.482660
Olubuse	004.558130	07.497465	48, Odi-Olowo street	004.572768	07.468603
Ajegunle	004.563083	07.497032	42, Mokuro road	004.569990	07.493120
Agric Area	004.573732	07.533373	50, Ile-Lami compound	004.565858	07.479435
Mount Zion	004.589283	07.498228			
Moore	004.566738	07.490868			
Eleyele	004.545620	07.490347			
Iredunmi	004.558257	07.482020			

**Table 2:** Proximity (300-meter buffer) from the ERFs to the cholera incidences.

ERFs	Total number of ERFs	Hot spots
Waste dump site	42	18
Market	18	7
Abattoir	34	2

Total	94	27

**Table 3:** Statistical relationship between ERFs and incidences of cholera.

ERF sites	Proximity to historic cholera cases	No proximity to historic cholera cases	Total	*χ* ^2^	*p* value
WDS	18 (40.9)	26 (59.1)	44		
Market	7 (38.9)	11 (61.1)	18	13.813	0.001^*∗*^
Abattoir	2 (5.6)	34 (94.4)	36		

Total	27 (27.6)	71 (72.4)	98		

*∗* indicates significance.
